# Molecular Techniques Complement Culture-Based Assessment of Bacteria Composition in Mixed Biofilms of Urinary Tract Catheter-Related Samples

**DOI:** 10.3389/fmicb.2019.00462

**Published:** 2019-03-20

**Authors:** Iva Kotaskova, Hana Obrucova, Barbora Malisova, Petra Videnska, Barbora Zwinsova, Tereza Peroutkova, Milada Dvorackova, Petr Kumstat, Pavel Trojan, Filip Ruzicka, Veronika Hola, Tomas Freiberger

**Affiliations:** ^1^Molecular Genetics Laboratory, Centre for Cardiovascular Surgery and Transplantation, Brno, Czechia; ^2^Medical Genomics Research Group, CEITEC, Masaryk University, Brno, Czechia; ^3^Department of Clinical Immunology and Allergology, Medical Faculty, Masaryk University, Brno, Czechia; ^4^Research Centre for Toxic Compounds in the Environment, Masaryk University, Brno, Czechia; ^5^Institute of Microbiology, Faculty of Medicine, Masaryk University and St. Anne's University Hospital, Brno, Czechia; ^6^Department of Urology, St. Anne's University Hospital, Brno, Czechia

**Keywords:** PCR-DGGE, urine culture, urinary catheter, ureteral catheter, double-J catheter, stent, biofilm, polymicrobial biofilm

## Abstract

Urinary or ureteral catheter insertion remains one of the most common urological procedures, yet is considered a predisposing factor for urinary tract infection. Diverse bacterial consortia adhere to foreign body surfaces and create various difficult to treat biofilm structures. We analyzed 347 urinary catheter- and stent-related samples, treated with sonication, using both routine culture and broad-range 16S rDNA PCR followed by Denaturing Gradient Gel Electrophoresis and Sanger sequencing (PCR-DGGE-S). In 29 selected samples, *16S rRNA* amplicon Illumina sequencing was performed. The results of all methods were compared. In 338 positive samples, from which 86.1% were polybacterial, 1,295 representatives of 153 unique OTUs were detected. Gram-positive microbes were found in 46.5 and 59.1% of catheter- and stent-related samples, respectively. PCR-DGGE-S was shown as a feasible method with higher overall specificity (95 vs. 85%, *p* < 0.01) though lower sensitivity (50 vs. 69%, *p* < 0.01) in comparison to standard culture. Molecular methods considerably widened a spectrum of microbes detected in biofilms, including the very prevalent emerging opportunistic pathogen *Actinotignum schaalii*. Using massive parallel sequencing as a reference method in selected specimens, culture combined with PCR-DGGE was shown to be an efficient and reliable tool for determining the composition of urinary catheter-related biofilms. This might be applicable particularly to immunocompromised patients, in whom catheter-colonizing bacteria may lead to severe infectious complications. For the first time, broad-range molecular detection sensitivity and specificity were evaluated in this setting. This study extends the knowledge of biofilm consortia composition by analyzing large urinary catheter and stent sample sets using both molecular and culture techniques, including the widest dataset of catheter-related samples characterized by *16S rRNA* amplicon Illumina sequencing.

## Introduction

Inserting urinary (so-called Foley) or Double-J catheters (DJC) is one of the most common urological interventions. At the same time, catheter-associated urinary tract infections (CAUTI) represent more than 40% of all nosocomial infections in healthcare units (Hooton et al., 2010). Although only a small part of generally colonized catheters develop into typically biofilm CAUTI, colonization is just enough to represent a risk for immunocompromised patients, cause financial loss, prolong patients' hospitalization, and impede clinical management. Despite this fact, urinary and ureteral catheter colonization has not been extensively studied yet, taking into account broad-range molecular techniques.

The bacterial composition of colonizing biofilm is affected by various factors such as indwelling time, sex, comorbidities, or patient conditions (Paick et al., 2003; Frank et al., 2009; Xu et al., 2012; Kliś et al., 2014). Gram-negative rods predominate on urinary catheters, while gram-positive cocci prevail on stents (Paick et al., 2003; Frank et al., 2009; Holá et al., 2010; Bonkat et al., 2011, 2012b, 2013; Choe et al., 2012; Xu et al., 2012; Kliś et al., 2014). Routine urine culture provides valuable quantitative information enabling us to distinguish between contamination/colonization and aetiological agents (Hooton et al., 2010), but results can be negatively influenced by known culture pitfalls.

There are various PCR-based techniques applicable to mixed samples (Choe et al., 2012; Xu et al., 2012). One of the most useful methods has been shown as Denaturing Gradient Gel Electrophoresis (DGGE), representing a non-high-throughput setting of polymicrobial sample analysis. Taxonomical characterization of separated amplicons is usually required in clinical samples and is possible by Sanger sequencing (Choe et al., 2012), alternatively in combination with chromatogram software separation by RipSeq Mixed tool (Kotásková et al., 2017). DGGE has been applied to different clinical materials (Davies et al., 2004; Li et al., 2005; Liu et al., 2015), but rarely to urinary catheter (Frank et al., 2009; Choe et al., 2012; Xu et al., 2012) or ureteral stents (Kliś et al., 2014). Modern Next-Generation Sequencing (NGS) techniques could be beneficial in studying highly diverse bacterial communities and/or in high-throughput study designs. High sensitivity and throughput demands make it suitable for research projects but not yet for single or few sample analysis in diagnostic laboratories.

In this study, we evaluated *16S rRNA* PCR-DGGE-S, and routine conventional culture's capability to determine the urinary and ureteral catheter biofilms' bacterial communities. The purpose of the study was to describe the bacterial composition of urinary and ureteral catheter biofilms and catheter-related samples and to compare the performance of culture and PCR-DGGE-S in a low scale setting, using *16S rRNA* Illumina sequencing as a reference method in selected specimens. To the best of our knowledge, we provide the most comprehensive urinary tract catheter-related specimen analysis using broad-range molecular techniques, while also addressing their sensitivity and specificity.

## Methods

### Sample Collection

During a study period from 2012 to 2014, in total 347 samples from 133 differentpatients (25.4% females) were collected, including urinary catheters (C), corresponding urine samples (CU), proximal and distal Double-J catheters tips (DJCP and DJCD), and corresponding Double-J catheter urine samples (DJCU), irrespective of the patient diagnosis or underlying disease, according to the collection strategy applied before (Xu et al., 2012), except of consecutive sampling approach (*n* = 155). Catheter removal was based on urologist decision. The study was approved by the Ethics Committee of the St. Anne's University Hospital in Brno. No informed consent was required because neither human cells nor human tissues were processed and no procedure in addition to standard care was performed.

Dataset characteristics, including patients' diagnoses, are shown in Table 1. During the urological intervention, the DJC or C was aseptically removed from the patient's body. The 5 cm long tips (proximal and distal part of DJC and the distal part of C) were snipped off for both anaerobic and aerobic culture. Equal parts were placed into sterile tubes containing 5 mL of Brain Heart Infusion (BHI) and Wilkins-Chalgren broth, respectively (Oxoid, UK). In parallel, urine samples obtained through the catheter before removal were also treated aseptically. Urine was voided in 18 sampling cases, because of patients' oligo/anuria in the time of collection. The samples were stored refrigerated and collected once a day for microbiological examination. The sonication procedure of BHI was described previously and consisted of two 5 min sonications interspaced by 2 min of vortexing (Holá et al., 2010). Sonication fluids and urines samples were used for inoculation and bacterial DNA extraction described below.

**Table 1:** Dataset characteristic and results overview.

	**C^a^**	**CU^b^**	**DJCP^c^**	**DJCD^d^**	**DJCU^e^**	**In total**
**SAMPLE CHARACTERISTICS**						
Collected samples	93	76	60	60	58	347
Complete material-related sets	75 doublets		57 triplets			132
Repeated samplings	18		2			20
**PATIENTS' DATA**						
Different patients (males/females)	74 (72/2)		59 (27/32)			133 (99/34)
Mean age ± SD^f^, (median)	76.7 ± 10.9 (62)		61 ± 14.8 (78.5)			69.7 ± 14.9 (72)
**DIAGNOSES**						
Prostatic or urinary tract cancer	25		3			28
Hydronephrosis	8		20			28
Urolithiasis without hydronephrosis	1		30			31
Acute cystitis	0		4			4
Prostatic hyperplasia	27		2			29
Urine retention^g^	13		0			13
**CULTURE RESULTS**						
Positive samples (% of analyzed)	92 (98.9)	73 (96.1)	48 (80)	46 (76.7)	19 (32.8)	278 (80.1)
Polybacterial samples (% of positive)	87 (94.6)	62 (84.9)	21 (43.8)	26 (56.5)	5 (26.3)	201 (72.3)
Isolates	330	190	80	87	28	715
Unique OTUs^h^	55	33	23	23	11	64
**PCR-DGGE-S^i^ RESULTS**						
Positive samples (% of analyzed)	93 (100)	76 (100)	55 (91.7)	52 (86.7)	55 (94.8)	331 (95.4)
Polybacterial samples (% of positive)	85 (91.4)	68 (89.5)	43 (78.2)	41 (78.8)	41 (74.5)	278 (84)
Detected representatives	283	201	138	147	145	914
Unique OTUs	58	59	63	63	59	118
**JOINED RESULTS ^j^ (CULTURE AND PCR-DGGE-S)**						
Positive samples (% of analyzed)	93 (100)	76 (100)	58 (99.7)	56 (93.3)	55 (94.8)	338 (97.4)
Polybacterial samples (% of positive)	90 (96.8)	71 (93.4)	43 (74.1)	46 (82.1)	41 (74.5)	291 (86.1)
Detected representatives	470	294	182	196	153	1295
Unique OTUs	87	70	73	73	61	153
**CULTURE AND PCR-DGGE-S COMPARISON**						
Concordantly identified bacteria (% of bacteria detected by culture or PCR-DGGE-S)	143 (30.4)	97 (33)	36 (19.8)	38 (19.4)	20 (13.1)	334 (25.7)
Samples with entirely concordant results (% of positive by culture or PCR-DGGE-S)	10 (10.8)	15 (19.7)	11 (18.9)	11 (19.6)	9 (16.4)	56 (16.6)
Samples with entirely discrepant results (% of positive by culture or PCR-DGGE-S)	61 (65.6)	42 (55.3)	25 (43.1)	22 (39.3)	4 (7.3)	154 (45.6)
Average Jaccard similarity index ± SD	0.37 ± 0.3	0.43 ± 0.34	0.27 ± 0.35	0.25 ± 0.32	0.21 ± 0.34	0.32 ± 0.34

a *C, catheters*;

b *CU, catheter urine*;

c *DJCP, proximal tip of double-J catheter*;

d *DJCD, distal tip of double-J catheter*;

e *DJCU, double-J catheter urine*;

f *SD, standard deviation*;

g *disabled patients, post-stroke condition, muscular dystrophy*;

h *OTU, operational taxonomic unit*;

i *PCR-DGGE-S, PCR denaturing gradient gel electrophoresis. Sanger sequencing*;

j *joined results are union of culture and PCR-DGGE-S result subsets*.

### Culture

The sonicated suspension and the urine samples (1 μL) were inoculated to a set of following solid media—Blood Agar (Columbia Blood Agar Base, Oxoid, United Kingdom; 7% of sterile sheep blood), UriSelect 4 (BioRad, France), Endo Agar (Imuna, Slovakia), Blood Agar supplemented with 10% of NaCl, Blood Agar supplemented with amikacin (32 mg/L), and Wilkins-Chalgren Agar (Wilkins-Chalgren Agar Base, Oxoid, United Kingdom; 7% of sterile sheep blood, LabMediaServis, Czech Republic; and vitamin K, Zentiva, Czech Republic).

The anaerobic cultivation was performed in an Anaerobic Work Station Concept 400 (Ruskinn Technology) for 7 days at 37°C with an atmosphere of 80% N_2_, 10% CO_2_, and 10% H_2_. The number of colonies was estimated after 48 h and 1 week in aerobic culture. Colony Forming Units (CFU) quantification was performed on Blood Agar; UriSelect helped with quantification of mixed cultures and their preliminary identification and isolation; other media were used for selective culture of given groups of microorganisms and for their preliminary identification. Endo Agar was used for culture of Gram-negative rods and Blood Agar supplemented with 10% of NaCl for the culture of staphylococci. Blood Agar supplemented with amikacin was used for culture of streptococci and Wilkins-Chalgren Agar for the culture of anaerobes.

All isolated strains were quantified and identified to the species/genus level biochemically (EN-COCCUStest, ENTEROtest 24, STAPHYtest 24, STREPTOtest 24, NEFERMtest 24 all Erba-Lachema, Czech Republic; API Coryne, API 20A, API 20Strep, API 20NE, all Biomerieux, France; RapID ONE System, RapID NF PLUS System, all Thermofisher Scientific, MA, USA). For verification of ambiguous results, matrix-assisted laser desorption/ionization time-of-flight mass spectrometry (MALDI-TOF MS) analysis, not fully implemented for routine use at the time of sample collection, was performed. MALDI Biotyper with FlexControl 3.4 software (Bruker Daltonik) was used according to manufacturer's instructions. The manufacturer-recommended cut-off scores were used for identification, with scores of ≥2.000 indicating identification to the species level, scores between 1.700 and 1.999 indicating identification to the genus level, and scores of <1.700 indicating no identification. Using this setting, bacteria in a quantity ≥10^3^ CFU/mL are routinely detected.

### PCR-DGGE-S

Sonication fluid (300–2,000 μL) and urine (1,000 μL) were centrifuged for 20 min/23,000 rpm and 10 min/14,000 rpm, respectively. Pellets were incubated with 130 μL lysis buffer, 20 μL lysozyme (180 mg/mL, Sigma-Aldrich, USA) and lysostaphin (1.8 mg/mL, Sigma-Aldrich, USA) for 30 min at 37°C. DNA was extracted by the QIAamp DNA Blood Mini Kit (Qiagen, Germany) according to the manufacturer's instructions.

The V3-V4 region of 16S rDNA (460 bp) was amplified using eubacterial primers FP338GC (Mrázek et al., 2008) and RP772 (Nadkarni et al., 2002). Amplification was carried out in a total volume of 35 μL of HotStarTaq Mastermix (Qiagen, Germany), 5 μL of template DNA, with 1.5 mM MgCl_2_, 0.5 μM of each primer and 0.16 mM 8-methoxypsoralen (8-MOP, Sigma-Aldrich, USA) concentration. Mixtures were incubated at 4°C for 1.5 h and exposed UVA (365 nm) for 7 min (30 J/cm^2^) for decontamination by the 8-MOP. PCR conditions were as follow: initial denaturation at 95°C for 15 min; 35 cycles of denaturation at 94°C for 30 s, primer annealing at 59°C for 1 min, extension at 72°C for 1 min followed by final extension at 72°C for 30 min to avoid artificial PCR products formation. PCR products were examined on 2% agarose gel stained with ethidium bromide. We were able to detect ~750 bacterial template copies entering PCR, roughly corresponding to a concentration of ~1.5·10^5^ template copies/mL.

PCR products were separated by DGGE using INGENYphorU-2x2 (Ingeny, The Netherlands) apparatus. DGGE was performed in 6% polyacrylamide (37:1 AA:BAA, Sigma-Aldrich, USA) with the 30–60% denaturing gradient (7 M urea and 40% formamide in 100% solution; Sigma-Aldrich, USA), in a 0.5xTAE running buffer. Gels were electrophoresed at a voltage of 12 V for 30 min, subsequently at 120 V for 15.5 h, at 60°C. Finally, the gel was stained with ethidium bromide for 20 min and documented. Visible bands were excised and eluted overnight in 50 μL of sterile water. Re-amplification was performed using forward primer without GC clamp. Products were visualized on 2% agarose gel, extracted from the gel by QIAquick Gel Extraction Kit (Qiagen, Germany) according to the manufacturer's protocol and sequenced with ABI PRISM 3130 Avant Genetic Analyser (Life Technologies, USA).

Re-amplified products were sequenced and compared with those in databases of RefSeq (Tatusova et al., 2014), SepsiTest-BLAST tool database (SepsiTest^TM^ BLAST)^1^ and 16SpathDB 2.0 (Identification of medically important bacteria by *16S rRNA* sequence).^2^ Sequence identity of ≥97 and ≥99% was required for genus and species identification, respectively; at least 0.5% difference between two different records was required for definite identification (Drancourt et al., 2000). Chimeras were checked with the DECIPHER tool (Wright et al., 2012).

### *16S rRNA* Amplicon Illumina Sequencing and Data Processing

*16S rRNA* amplicon MiSeq Illumina sequencing was used as a reference method to evaluate PCR-DGGE-S and culture. Because of the study budget restrictions, 30 random samples were selected for NGS analysis. To reflect a different level of PCR-DGGE-S and culture results concordance, a Jaccard similarity index (*J*; *J* = 0 refer to complete discordance, *J* = 1 to complete concordance) was applied. Twenty-nine samples (10 with *J* = 0; 10 with *J* = 1, and 9 with 0.25 ≤ *J* ≤ 0.5) with sufficient results' quality were used for the next analyses.

The V3-V4 region of 16S rDNA was targeted by PCR using the barcoded primers (Klindworth et al., 2013). Products were cleaned-up using AMPure magnetic beads (Beckman Coulter, USA) and concentration measured by Qubit HS. Samples were pooled and indexing reactions with KAPA HiFi HotStart ReadyMix and Nextera primers (Illumina, USA) were performed. Products were cleaned by AMPure beads, and precise template concentration was determined by KAPA Library Quantification Kit. Prepared libraries were sequenced by MiSeq (Illumina, USA) using V3 Illumina kit, resulting to 150 bp pair-end reads.

Pair-end reads passing quality control were merged using the fastq-join method in QIIME 1.9.1 (Caporaso et al., 2010). Data were demultiplexed, barcodes and primers were trimmed in R. OTUs (Operational Taxonomic Units) were constructed as clusters of >97% sequence similarity using QIIME. Chimeras were detected with UCHIME in USEARCH v6.1.544 (Edgar et al., 2011) and excluded. Taxonomy was assigned to each OTU based on SILVA 123 reference database (Pruesse et al., 2007).

### Statistical Analysis

Richness as a number of OTUs per sample was assessed for α-diversity evaluation. Pair *t*-test and repeated-measures ANOVA with *post-hoc* Tukey test and Bonferroni corrections for multiple hypothesis testing were used to test clinical material's effect on richness and Jaccard index in complete catheter-related pairs and complete Double-J catheter-related triplets, respectively. Fishers' exact test was employed to evaluate the importance of Gram-positive and Gram-negative representatives. We have tested OTUs association with patients' diagnosis (listed in Table 1), interspecies relations and tending of single OTUs to mono- vs. polybacterial occurrence by Chi-Square test with Yate's correction. A patient was considered positive, if a bacterium was present in at least one of his/her samples. Species with low prevalence were merged together and OTUs according to genera were created. Only those OTUs with frequency higher than 5 were included.

To estimate β-diversity with a lack of abundance data, we assessed the Shannon index based on genera detection frequencies by particular methods in each clinical material type. PCA was performed with a species-trait matrix and covariance biplots were constructed using Past v3.15. (Ryan et al., 2001) The six most prevalent OTUs detectable by culture as well as PCR-DGGE-S were intentionally chosen for analytical parameter evaluation. McNemar's tests with continuity correction were performed reciprocally. Specificity and sensitivity were defined for culture (PCR-DGGE-S as a reference method) and PCR-DGGE-S (culture as a reference method) separately, as used before (Zijnge et al., 2006) and tested by pair *t*-test. All null hypotheses were rejected at α ≥ 0.05, the lower significance levels are specified in the text.

## Results

**Figure 1:**
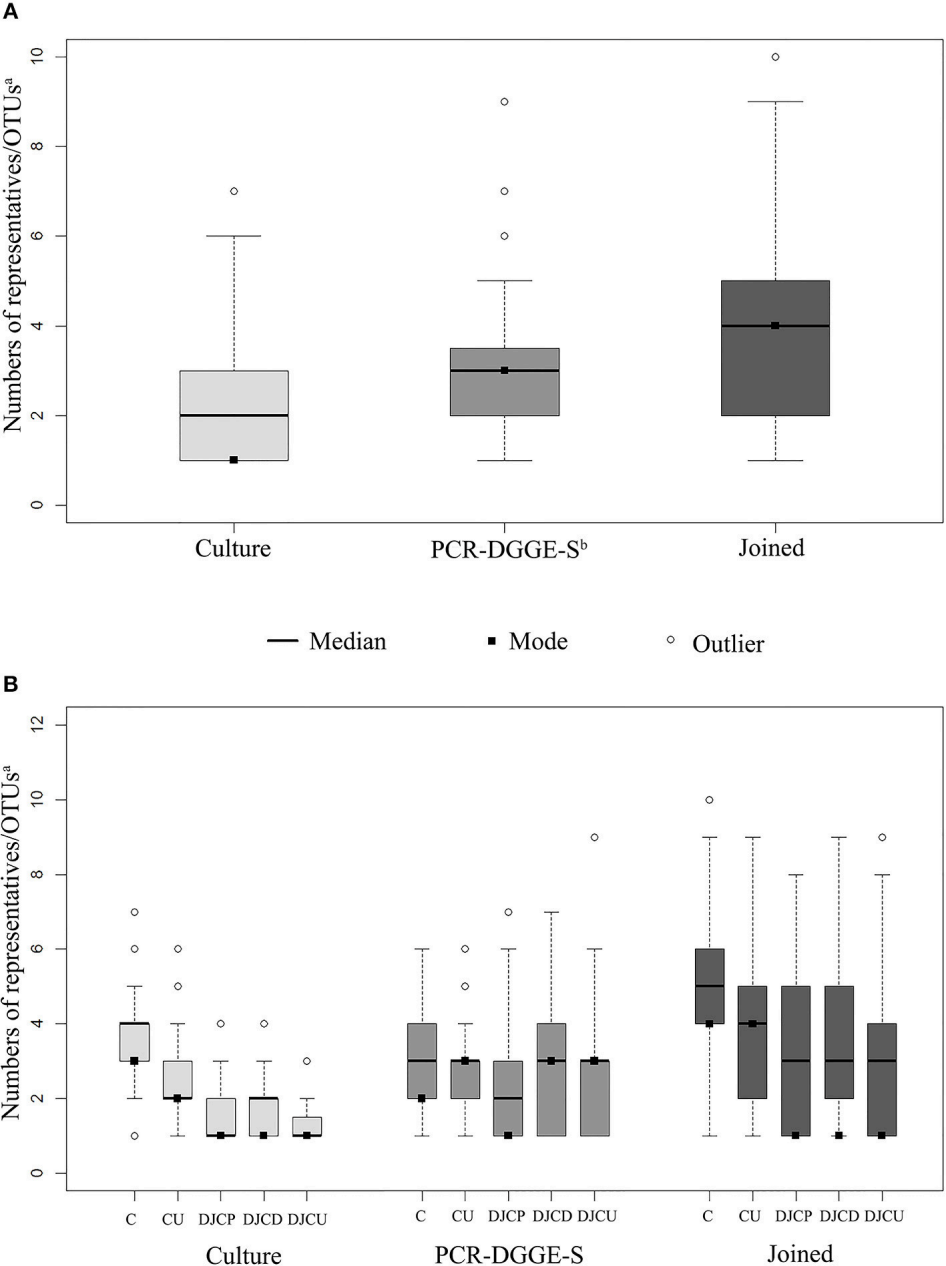
Sample boxplots. Graph **(A)** shows the distribution in sample results regarding methods, **(B)** shows results distribution regarding the method and material type. Joined results are the union of culture and PCR-DGGE-S result subsets. The whiskers represent 1.5 interquartile range. ^a^OTU, operational taxonomic unit; ^b^PCR-DGGE-S, PCR denaturing gradient gel electrophoresis, Sanger sequencing; C, catheters; CU, catheter urine; DJCP, proximal tip of double-J catheter; DJCD, distal tip of double-J catheter; DJCU, double-J catheter urine.

A culture revealed 715 isolates in 278 positive samples (80.1% positivity rate), while PCR-DGGE-S was able to detect 334 positive samples (95.4% positivity rate) with 914 representatives, for details see Figure 1. PCR-DGGE did not separate mixed amplicons in 40 samples. Nine sequences remained unassessed to any OTU after chimera filtration. Details on culture and PCR-DGGE-S results are reported in Tables 1, 2, predominantly represented families are in Table 3.

**Table 2:** Frequencies of detection regarding material and method.

	**Culture**						**PCR-DGGE-S^a^**						**Joined results^b^**					
**OTU^c^**	**C^d^**	**CU^e^**	**DJCP^f^**	**DJCD^g^**	**DJCU^h^v**	**In total**	**C**	**CU**	**DJCP**	**DJCD**	**DJCU**	**In total**	**C**	**CU**	**DJCP**	**DJCD**	**DJCU**	**In total**
*Abiotrophia*	1					1							1					1
Acinetobacter^*^	1	1		1		3	1	1	3	3		8	1	1	3	4		9
*Actinotignum*							37	21	14	14	11	97	37	21	14	14	11	97
*Actinomyces*							2	2	1	3	2	10	2	2	1	3	2	10
*Aerococcus*	5					5	10	8	1	1	1	21	12	8	1	1	1	23
*Alcaligenes*	5	2				7	1	1				2	5	3				8
*Alloscardovia*							1		1	2	2	6	1		1	2	2	6
*Anaerococcus*							2	1	1			4	2	1	1			4
*Atopobium*							1		1	1	1	4	1		1	1	1	4
*Bacteroides*									1	1	2	4			1	1	2	4
*Bifidobacterium*							3	1	6	5	7	22	3	1	6	5	7	22
*Burkholderiales*							1					1	1					1
*Campylobacter*							12	9	7	7	5	40	12	9	7	7	5	40
*Cellulosimicrobium*										1	1	2				1	1	2
*Citrobacter*	11	7	1	2		21	8	7	2	2		19	13	9	2	3		27
*Corynebacterium*	1	1				2	2	5	6	5	16	34	2	5	6	5	16	34
*Cronobacter*	1	1				2	1	2				3	1	2				1
*Dialister*											1	1					1	1
*Dolosigranulum*									1			1			1			1
Enterobacter^*^	9	6				15	11	10	1			22	12	10	1			25
*Enterobacteriaceae*	1	1				2	2	1			1	4	2	1			1	4
Enterococcus^*^	74	49	16	20	7	166	44	31	13	16	9	113	77	52	20	24	11	184
Escherichia^*^	43	30	14	17	7	111	30	24	6	9	10	79	46	33	15	19	11	124
*Facklamia*	1					1							1					1
*Fastidiosipila*							4	1		1		6	4	1		1		6
*Finegoldia*									1	2	2	5			1	2	2	5
*Firmicutes*								1				1		1				1
*Fusobacterium*							5	4	4	2	5	20	5	4	4	2	5	20
*Gardnerella*							1		3	5	7	16	1		3	5	7	16
*Kingella*	1					1							1					1
Klebsiella^*^	36	19	10	10	4	79	9	5	7	5	5	31	37	19	11	10	6	83
*Kocuria*	2					2			1	1	2	4	2		1	1	2	6
*Lactobacillus*							3	3	4	5	6	21	3	3	4	5	6	21
*Lactococcus*											1	1					1	1
*Leptotrichia*									1	2	1	4			1	2	1	4
*Mobiluncus*									1	1	2	4			1	1	2	4
*Moraxella*							1	1	1	1		4	1	1	1	1		4
Morganella^*^	9	4	3	3	1	20	4	4	1	3	2	14	13	8	4	5	3	33
*Neisseria*							1	1		1		3	1	1		1		3
*Nocardiales*										1		1				1		1
*Oligella*								2				2						2
*Pantoea*	1					1	1	1				2	2	1				3
*Parviromonas*							1		1	1		3	1		1	1		3
*Peptoniphilus*							1		5	4	5	15	1		5	4	5	15
*Porphyromonas*									1	2	1	4			1	2	1	4
*Prevotella*	2		2	2		6			4	2	2	8	2		5	3	2	12
*Propionibacterium*							1	2	5	3	5	16	1	2	5	3	5	16
*Propionimicrobium*							25	11	6	7	3	52	25	11	6	7	3	52
Proteus^*^	44	28	3	6	1	82	22	15	4	5	1	47	46	29	5	10	2	92
*Providencia*	7	7	3	1	1	19	8	7	2			17	10	9	4	1	1	25
Pseudomonas^*^	19	11	4	5	2	41	7	9	3	4	3	26	20	16	5	7	4	52
*Raoultella/Citrobacter*							1	1				2	1	1				2
*Serratia*	11	3				14	1				1	2	11	3			1	15
*Staphylococcus*	36	19	19	15	4	93	3	2	1	2	2	10	27	17	12	10	2	68
CoNS^i^^*^							12	3	8	7	11	41	12	3	8	7	11	41
*Stenotrophomonas*	1					1			1			1	1		1			2
*Streptococcus*	8	1	5	5	1	20	1	2	5	5	7	20	9	3	9	9	7	37
*Varibaculum*										1	1	2				1	1	2
*Veillonella*							2	2	1	1	1	7	2	2	1	1	1	7
*Williamsia*									2	3		5			2	3		5
**In total**	**330**	**190**	**80**	**87**	**28**	**715**	**283**	**201**	**138**	**147**	**145**	**914**	**470**	**294**	**182**	**196**	**153**	**1295**
Gram-negative	202	120	40	47	16	425	130	107	54	56	50	397	247	162	76	85	56	628
(% of detected representatives)	61.2	63.2	50.00	54	57.1	59.4	46	53.2	39.1	38.1	34.5	43.4	52.6	55.1	41.8	43.4	36.6	48.5
Number of rare OTUs^j^	9	5	1	2	4	5	16	11	19	13	12	7	16	10	17	14	12	10
(% of detected OTUs)	36	29.4	9.1	16.7	44.4	20	40	31.4	46.3	31.7	32.4	12.3	34.8	28.6	41.5	33.3	31.9	16.7

a *PCR-DGGE-S, PCR denaturing gradient gel electrophoresis, Sanger sequencing*;

b *Joined results are union of culture and PCR-DGGE-S result subsets*;

c *OTU: operational taxonomic unit*;

d *C, catheters*;

e *CU, catheter urine*;

f *DJCP, proximal tip of double-J catheter*;

g *DJCD, distal tip of double-J catheter*;

h *DJCU, double-J catheter urine*;

i *CoNS, coagulase-negative staphylococci*.

j *Singletons regarding result set and material type are considered as rare OTUs. Frequencies higher than 1 are shown in a gray heat map, darker gray indicates higher frequency. Asterisk indicates common genera defined in the literature (Azevedo et al., 2017)*.

**Table 3:** Five most prevalent families regarding the material and result set in decreasing prevalence.

	**Culture**	**PCR-DGGE-S^a^**	**Joined results^b^**
C^c^	*Enterobacteriaceae*	*Enterobacteriaceae*	*Enterobacteriaceae*
	*Enterococcaceae*	*Enterococcaceae*	*Enterococcaceae*
	*Staphylococcaceae*	*Actinomycetaceae*	*Actinomycetaceae*
	*Pseudomonadaceae*	*Propionibacteriaceae*	*Staphylococcaceae*
	*Streptococcaceae*	*Staphylococcaceae*	*Propionibacteriaceae*
CU^d^	*Enterobacteriaceae*	*Enterobacteriaceae*	*Enterobacteriaceae*
	*Enterococcaceae*	*Enterococcaceae*	*Enterococcaceae*
	*Staphylococcaceae*	*Actinomycetaceae*	*Actinomycetaceae*
	*Pseudomonadaceae*	*Propionibacteriaceae*	*Staphylococcaceae*
	*Alcaligenaceae*	*Campylobacteraceae*	*Pseudomonadaceae*
DJCP^e^	*Enterobacteriaceae*	*Enterobacteriaceae*	*Enterobacteriaceae*
	*Staphylococcaceae*	*Actinomycetaceae*	*Enterococcaceae*
	*Enterococcaceae*	*Enterococcaceae*	*Staphylococcaceae*
	*Streptococcaceae*	*Propionibacteriaceae*	*Actinomycetaceae*
	*Pseudomonadaceae*	*Bifidobacteriaceae*	*Propionibacteriaceae*
DJCD^f^	*Enterobacteriaceae*	*Enterobacteriaceae*	*Enterobacteriaceae*
	*Enterococcaceae*	*Actinomycetaceae*	*Enterococcaceae*
	*Staphylococcaceae*	*Enterococcaceae*	*Actinomycetaceae*
	*Pseudomonadaceae*	*Bifidobacteriaceae*	*Staphylococcaceae*
	*Streptococcaceae*	*Propionibacteriaceae*	*Bifidobacteriaceae*
DJCU^g^	*Enterobacteriaceae*	*Enterobacteriaceae*	*Enterobacteriaceae*
	*Enterococcaceae*	*Actinomycetaceae*	*Actinomycetaceae*
	*Staphylococcaceae*	*Bifidobacteriaceae*	*Bifidobacteriaceae*
	*Pseudomonadaceae*	*Corynebacteriaceae*	*Corynebacteriaceae*
	*Streptococcaceae*	*Staphylococcaceae*	*Staphylococcaceae*
**Total results**	*Enterobacteriaceae*	*Enterobacteriaceae*	*Enterobacteriaceae*
	*Enterococcaceae*	*Actinomycetaceae*	*Enterococcaceae*
	*Staphylococcaceae*	*Enterococcaceae*	*Actinomycetaceae*
	*Pseudomonadaceae*	*Propionibacteriaceae*	*Staphylococcaceae*
	*Streptococcaceae*	*Staphylococcaceae*	*Propionibacteriaceae*

a *PCR-DGGE-S, PCR denaturing gradient gel electrophoresis, Sanger sequencing*;

b *Joined results are union of culture and PCR-DGGE-S result subsets*;

c *C: catheters*;

d *CU: catheter urine*;

e *DJCP, proximal tip of double-J catheter*;

f *DJCD, distal tip of double-J catheter*;

g *DJCU, double-J catheter urine*.

### Joined Culture and PCR-DGGE-S Results

Joined culture and PCR-DGGE-S subset results indicated 338 positive samples (97.4% of analyzed) with 1,295 identified representatives (details in Figure 1 and Tables 1, 2). Forty-seven samples (13.9% of positive) were mono-microbial. In total, we identified 101 unique species (55 genera of 30 different families). Joined results revealed 470 (42 genera of 22 families) and 239 (46 genera of 26 families) representatives in C and DJC sonicates regardless the tip origin. In DJC proximal and distal parts, 182 (40 genera of 23 families) and 196 (40 genera of 24 families) representatives were identified, respectively. Urine samples contained 294 (33 genera of 19 families) and 153 (36 genera of 22 families) representatives in CU and DJCU, respectively. Predominantly detected families are in Table 3.

C and CU results showed exact taxonomic concordance in all detected species for 24 doublets (32% of complete doublets). Concordant DJCP and DJCD taxonomic results were recorded in 21 triplets (36.8% of complete triplets); 11 of those were concordant in all three materials (19.3% of complete triplets). No significant difference in richness was observed among DJC-related samples (comparing DJCD, DJCP, DJCU), while a higher richness was shown in C than CU (*p* < 0.01) as well as comparing DJC regardless the tip and DJCU (*p* < 0.01). Higher richness mean was shown in C (5.05) than DJC regardless the tip origin (3.95) (*p* < 0.002). A significantly higher ratio of Gram-positive representatives in DJC-related samples compared to catheter-related samples was obvious (*p* < 0.00001, for details, see Table 2).

We have observed a higher *Gardnerella vaginalis* (*p* < 0.01) and *Klebsiella* spp. (*p* < 0.01), but lower *Enterococcus* spp. (*p* < 0.01) prevalence in patients with urolithiasis compared to patients without this diagnosis. Further, we observed mutually antagonistic occurrence of *Enterococcus* spp. and *G. vaginalis* (*p* < 0.01) while co-occurrence of *Enterococcus* spp. and *Escherichia coli* (*p* < 0.01), *Actinotignum schaalii* and *Propionimicrobium lymphophilum* (*p* < 0.01), *Fusobacterium nucleatum* and *Streptococcus* spp. (*p* < 0.01), and *G. vaginalis* together with *Lactobacillus* spp. (*p* < 0.01) (see Table S1).

Several OTUs [*Proteus* spp. (*p* < 0.01), *E. coli* (*p* < 0.01), *Enterococcus* spp. (*p* < 0.01), *Klebsiella* spp. (*p* < 0.05), and coagulase-negative *Staphylococci* (*p* < 0.05)] significantly preferred polybacterial rather than monomicrobial environment while opposite preference was not found in any OTUs.

### Comparison of Culture and PCR-DGGE-S

One hundred and sixty representatives were detected by PCR-DGGE-S in 60 culture-negative samples, while 9 isolates were cultured in 6 PCR negative samples; 9 specimens were concordantly negative using both methods. In total, 581 OTUs in 259 samples were not detected by culture, but by PCR-DGGE-S, while 382 isolates in 185 samples were not identified by PCR-DGGE-S, but by culture. A significantly higher proportion of Gram-positive bacteria was detected by PCR-DGGE-S than culture in DJCD and DJCU (both *p* < 0.05), as well as C and CU (both *p* < 0.0002).

We observed a statistically higher overall richness and rare genera resulting in a higher Shannon index using PCR-DGGE-S (*p* < 0.01) (see Table 2 and Figure S1). When focused on the particular clinical material's community structure, a deflection of catheter communities identified by culture was apparent, while communities identified by PCR-DGGE-S were grouped into two (C-related and DJC-related) groups (see Figure S2). When a Jaccard similarity index (evaluating α diversities, thus method agreement) was compared, a significantly higher mean for CU than DJCU (*p* < 0.05) was apparent, referring to more concordant results obtained by both methods in the CU than DJCU sub-dataset. Other significant differences were not observed.

**Figure 2:**
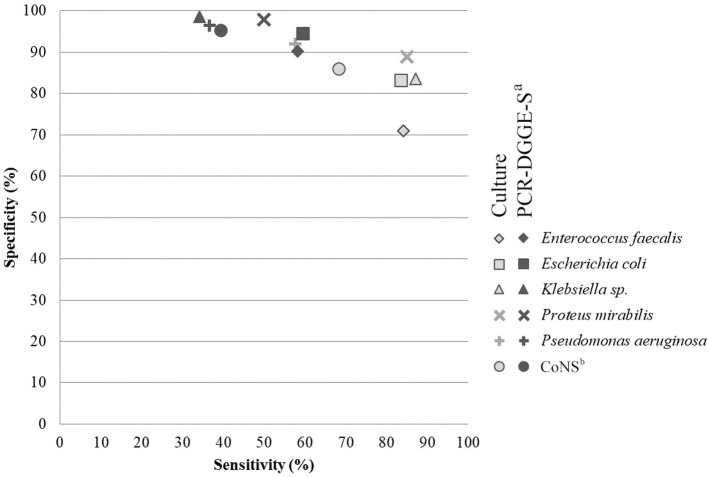
Comparison of analytical sensitivity and specificity of culture and PCR-DGGE-S. The figure shows analytical specificity and sensitivity relation. Parameters for six most prevalent species detectable by both methods were chosen and included. Light-gray indicates values for culture (PCR-DGGE-S is a reference method) and dark-gray for PCR-DGGE (culture is a reference), respectively. ^a^PCR-DGGE-S, PCR, denaturing gradient gel electrophoresis, Sanger sequencing; ^b^CoNS, Coagulase-negative Staphylococci.

To evaluate the sensitivity and specificity of broad-range detection, we selected data for the 6 most frequently detected OTUs (see Figure 2 and Table 4). The overall higher culture sensitivity (69 vs. 50%, *p* < 0.01) and PCR-DGGE-S specificity (95 vs. 85%, *p* < 0.01) was noted.

**Table 4:** Analytical parameters (%) of culture (A.) and PCR-DGGE-S (B.) for six most prevalent OTUs regarding the material and method.

	**C^a^**		**CU^b^**		**DJCP^c^**		**DJCD^d^**		**DJCU^e^**	
	**Sensitivity**	**Specificity**	**Sensitivity**	**Specificity**	**Sensitivity**	**Specificity**	**Sensitivity**	**Specificity**	**Sensitivity**	**Specificity**
**(A)**										
*Enterococcus faecalis*	**93**	**37**	**91**	**55**	69	85	73	82	56	96
*Escherichia coli*	**90**	**75**	88	87	**83**	**83**	**78**	**80**	60	98
*Klebsiella* sp.	**89**	**67**	**100**	**80**	86	92	100	91	60	98
*Proteus mirabilis*	**85**	**78**	75	83	50	98	25	91	NA	98
*Pseudomonas aeruginosa*	**86**	**85**	50	90	67	96	50	95	33	98
CoNS^f^	**83**	**78**	**67**	**88**	**88**	**83**	75	87	**25**	**100**
Average	88	70	78	80	74	90	67	88	39	98
**(B)**										
*Enterococcus faecalis*	**57**	**86**	**59**	**89**	56	91	58	90	71	92
*Escherichia coli*	**63**	**94**	70	95	**36**	**98**	**41**	**95**	86	92
*Klebsiella* sp.	**22**	**98**	**26**	**100**	60	98	50	100	75	96
*Proteus mirabilis*	**52**	**95**	45	95	67	96	17	94	NA	98
*Pseudomonas aeruginosa*	**32**	**99**	36	94	50	98	40	96	50	96
CoNS	**36**	**97**	**18**	**98**	**44**	**98**	46	96	**100**	**84**
Average	43	95	43	95	52	97	42	95	64	93

a *C, catheters*;

b *CU, catheter urine*;

c *DJCP, proximal tip of double-J catheter*;

d *DJCD, distal tip of double-J catheter*;

e *DJCU, double-J catheter urine*;

f *CoNS, Coagulase-negative staphylococci. Table (A) shows sensitivity (%) and specificity (%) of culture (PCR-DGGE-S was a reference method), whereas (B) shows sensitivity (%) and specificity (%) of PCR-DGGE-S (culture was a reference method). Statistically significant differences (McNemar's test with continuity correction) in comparison to reference method are marked in bold (p < 0.05)*.

### Comparison to *16S rRNA* Amplicon Illumina Sequencing

Illumina sequencing targeting the 16S rDNA's V3-V5 region detected 274 representatives in 29 samples with an average sequencing depth of 4,515 reads per sample. Only OTUs representing ≥0.1% of samples' total reads were included in the analyses.

**Figure 3:**
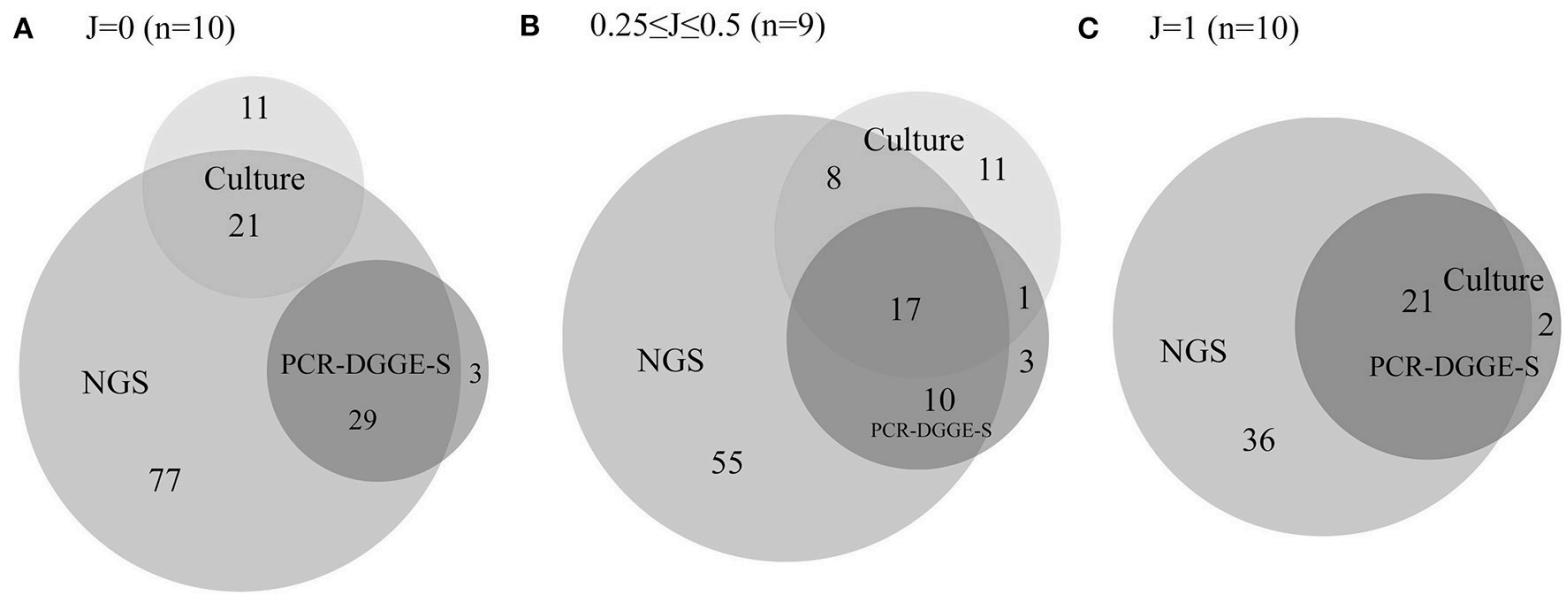
Euler diagrams with a number of representatives detected by culture, PCR-DGGE-S and NGS. The figure shows Euler proportional diagrams with absolute numbers of representatives detected by particular methods. Diagram **(A)** shows samples with no concordance in culture and PCR-DGGE-S results, therefore these sets are not overlapping. Diagram **(B)** shows samples with partial concordance in culture and PCR-DGGE-S results, and finally diagram **(C)** shows samples with entirely concordant culture and PCR-DGGE-S results, therefore dark set represents results of both these methods. In 17 samples, PCR-DGGE-S or culture identified additional representatives over the NGS, while NGS detected an additional bacteria over any non-NGS method in 28 out of 29 sample.

In 29 evaluated samples, NGS confirmed the presence of 106 from 137 representatives detected by culture or PCR-DGGE-S. NGS did not demonstrate the presence of 31 representatives. Twenty-two of them were detected solely by culture, not by NGS or PCR-DGGE-S (8 *Klebsiella* spp., 4 *Staphylococcus* spp., others with ≤ 2 occurrence, details are in Tables S2–S4), compared to 6 OTUs positive only by PCR-DGGE-S and not by NGS or culture (3 *P. lymphophilum*, others with single occurrence) and thus could be considered falsely positive. Another 3 were by detected by both culture and PCR-DGGE-S (*Citrobacter freundii, Proteus vulgaris*, and *Klebsiella oxytoca*), and therefore could be regarded as falsely negative NGS results (1.1 % of OTUs identified by NGS). NGS revealed an additional 168 representatives (122.6% of those identified by other 2 methods). For details in particular sample sets, see Tables S2–S4. For Euler diagrams representing the contribution of a particular method, see Figure 3.

## Discussion

Overall bacterial colonization is reported in the literature lower on DJC than C: compare 2.2–25% (Ozgur et al., 2013) to 5–95% (Maki and Tambyah, 2001) depending on the indwelling time. Our results showed an overall lower positivity rate of DJC-related samples in comparison to catheter-related samples (32.8–80 vs. 96.1–98.9% using culture and 86.7–94.8 vs. 100% using PCR-DGGE-S). PCR-DGGE-S detected more polybacterial samples than culture in each material except C. This was probably caused by preferential Gram-positive taxon detection by PCR-DGGE-S over all materials. The number of mono-bacterial samples was considerably higher in DJC than C (*p* < 0.00001), and in DJCU than CU (*p* < 0.02). These findings are not surprising, taking into account the location of the analyzed material in the body. The DJC is inserted into a primarily sterile body site, therefore the colonization of such DJC takes longer and has lower diversity. Thus, the number of mono-microbial biofilms or even negative samples is higher in DJC (and so DJCU) than C (and so CU) inserted into the more often colonized urethra.

Focusing on C and CU, exact taxonomic concordance was observed in 32% of doublets, Xu et al. (2012) observed concordance in 3 out of 14 patients (21.4%) using culture techniques and even lower concordance (12.5%) employing PCR-DGGE for interior vs. lumen of the same catheter. We report concordant results in 19.3% of DJC-related triplets. Compare this to Ozgur et al. (2013), reporting concordant taxonomic results in urine and DJC in just 1 out of 10 patients (10%).

The overlap of catheter and urine results from the same patient was relatively high in our samples compared to the other studies. Still there are many discrepancies, which may be explained as follows. If the microbe is identified in the catheter but not the urine, we assume the effect of antibiotic therapy (mainly in DJC samples) killing planktonic cells but not those embedded in the biofilm. Low cell concentration in biofilm not exceeding critical level can preclude the detachment of cells to urine. Moreover, species consortia composition is heterogeneous on the proximal and distal catheters' parts, as well as lumen and the outer surface (Frank et al., 2009; Xu et al., 2012). Therefore, urine microbiota can be affected by the lumen, but not the exterior colonization. Exterior biofilm should be more diverse and extensive because bacteria ascend through the catheter-urethral interface extraluminally (66%) more often than intraluminally (34%) (Tambyah et al., 1999). On the other hand, when a microbe is not detected from the catheter but urine, we assume bacterial feature contribution, such as higher bacterial adherence to epithelial cells than artificial/already colonized surfaces, or higher occurrence in the planktonic state rather than biofilm (Reid et al., 2011). Transient bacteriuria is another possible explanation.

### Urinary Catheter and Related Urine Samples Positivity Rates

Our study showed a high urinary catheter colonization positivity rate, detected by both PCR-DGGE-S and culture (100 and 98.9%). Xu et al. (2012) achieved a comparable positivity rate on catheters by culture (95.8%), however, a much lower rate by molecular techniques (58.3%). Simultaneously, these authors reported a much lower positivity rate in urine compared to catheters using culture (43.8 vs. 95.8%), while we achieved similar positivity rates in urine and catheter sonicates using both culture and PCR-DGGE-S (96.1 vs. 98.9% and 100 vs. 100%, respectively). These different results might be caused by different urine processing. Of note, they applied Maki's roll-plate technique prior to forceful scraping, which could cause an enhanced transfer of bacterial cells to an agar plate prior to sonication, leading to a significantly lower detection rate by molecular methods. Published studies on preferred pre-analytical technique are conflicting (Bonkat et al., 2011, 2012a). We suggest that catheter or stent sonication better ensures unified material with a higher number of intra- and extra-luminar colonizers entering both culture and molecular analysis and decreases the risk of contamination.

### Double-J Catheter and Related Urine Samples Positivity Rates

Our results, showing a higher positivity rate of DJC sonication fluids captured by PCR-DGGE than by culture (91.7 vs. 78.3%), are consistent with other studies. Kliś et al. (2014) even found 100% colonization incidence employing PCR-DGGE. Bonkat et al. (2011), Paick et al. (2003), and Farsi et al. (1995) reported 36, 44, and 68% culture positivity rate in DJC sonicates, respectively.

In DJCU, we identified 94.8 and 32.8% positive samples by PCR-DGGE-S and culture, respectively. Though DJCU culture positivity rate could seem low, it corresponds to other authors' results. Farsi et al. (1995) detected 29.9%, Paick et al. (2003) 21%, and Kliś et al. (2014) just 13% positive urine cultures.

An apparently lower positivity rate associated with very low richness was noted in DJCU samples using culture compared to molecular techniques. This can be attributed to antibiotic prophylaxis in patients undergoing the stent replacement, easily affecting the planktonic bacterial cell viability in urine samples, letting the viability of biofilm-embedded bacteria unaffected, at the same time. Moreover, device colonization is not necessarily manifested by bacteriuria (Paick et al., 2003; Xu et al., 2012; Ozgur et al., 2013), especially when Gram-positive colonizers are present (Kliś et al., 2014). On the contrary, it could not be explained simply by the presence of fastidious and uncommon bacteria in samples which would more likely be detected by the molecular method. In fact, typical uropathogens such as *E. faecalis* and *E. coli* were identified more often by PCR-DGGE-S than culture just in this material.

### Culture and PCR-DGGE-S Analytical Parameters

The gold standard in CAUTI diagnostics is quantitative culture. Few studies evaluating an analytical performance of PCR-based approach applied on urine or sonication fluid samples have been published. However, all of them were based on multiplex qPCR (Lehmann et al., 2010; Hansen et al., 2013; van der Zee et al., 2016), while broad-range assays were neglected. In this study, we present broad-range bacterial detection's analytical performance. For analytical parameters evaluation, we used results of mostly detected species identifiable by both methods. Therefore, no technique-related bias should affect sensitivity and specificity evaluation.

In general, the culture techniques registered significantly higher sensitivity than PCR-DGGE-S (69 vs. 50%, *p* < 0.01) while PCR-DGGE-S showed significantly higher specificity than culture techniques (95 vs. 85%, *p* < 0.01). Interestingly, in cases of catheter sonication fluid, a statistically significant difference in sensitivity and specificity between both methods was observed for each of the evaluated pathogens.

Our average PCR-DGGE-S specificity (95%) was higher than those obtained by other authors (83–90%). Four hundred and sixty bp long region of 16S rDNA sequencing can cause higher specificity than qPCR identification based on the complementarity of shorter species-specific multiple probes designed for 16S rDNA. On the other hand, our PCR-DGGE-S analytical sensitivity (49%) is much lower than the reported values of 81–100% (Lehmann et al., 2010; Hansen et al., 2013; van der Zee et al., 2016). We assume that lower PCR-DGGE-S sensitivity in easy to grow pathogens, in all materials but DJCU discussed above, is partly related to a higher detection threshold (1.5·10^5^ copies of template/mL) than culture (10^3^ CFU/mL). Moreover, a broad-range approach is well-known for multi-template PCR phenomenon, when the particular species sensitivity may decrease due to the competitive inhibitory effect of considerably un-equimolar DNA proportions entering the PCR (Kanagawa, 2003), emphasized by DGGE itself (Muyzer et al., 1993). Another un-equimolar template proportion issue has been reported when employing Sanger sequencing. Exceeding 1:10 concentration ratio can result in outcompeting the lower concentration template by the higher one and can make a lower concentrated PCR-DGGE co-migrated amplicons invisible on sequencing chromatogram (Kommedal et al., 2009).

Another reason for lower PCR-DGGE-S sensitivity might reside in a possible culture over-detection. Although we realize molecular-based technique limits including ability to detect DNA of non-living bacteria, and the risk of contamination (Salter et al., 2014), when using NGS as a reference and superior sensitive method, culture seemed to over-detect some bacteria considerably more than PCR-DGGE-S. It is demonstrated by the fact that NGS did not detect 22 OTUs positive by culture (8 *Klebsiella* sp., 4 *Staphylococcus* sp., others with ≤ 2 occurrence) compared to 6 OTUs positive by PCR-DGGE-S (3 *P. lymphophilum*, others with single occurrence) in selected entirely and partially discrepant samples as assessed by culture and PCR-DGGE-S (see also Figure 3). Thus, a suspected culture over-detection and contamination would make an overall relatively higher sensitivity achieved by culture than PCR-DGGE-S at least partly artificial. Coagulase-negative *Staphylococci* can be regarded as a common contaminant. On the other hand, the potential contamination issue during culture technique cannot explain a higher culture detection rate of e.g., *Klebsiella* sp., *E. coli* or *Staphylococcus aureus*. Both PCR-DGGE-S and culture, but not NGS detected *P. vulgaris, K. oxytoca* and *C. freundii* in 3 samples. Other *Enterobacteriaceae* family species, as well as unspecified reads belonging to this family were detected by NGS in these samples. Therefore, we assume contribution from the problematic *16S rRNA* sequence-based *Enterobacteriaceae* family taxonomy to these NGS false negative results, as well as lower *Klebsiella* sp. identification sensitivity by both PCR-based techniques.

Easy-growing bacteria might contribute to true higher culture sensitivity even if present in low quantity and/or if exceeded at least 10-fold by other strains, while PCR-DGGE-S often fails in these situations (Muyzer et al., 1993). It is supported by the fact that culture and NGS positive and PCR-DGGE-S negative were most commonly *E. faecalis* and *E. coli*. Six of 9 cases of *E. faecalis* had <5% abundance by NGS and one of 22 culture positive samples remained NGS-negative. Three of 5 *E*. *coli* had <2% abundance, and 1 out of 15 *E. coli* culture positive samples remained NGS-negative.

DJCU samples reflected a specific circumstance. We assume that lower culture rather than higher PCR-DGGE-S sensitivity (compare 39 and 64%) caused by prophylactic antibiotic administration might be a reason why DJCU is the only material where easy to grow uropathogens were identified more frequently by PCR-DGGE-S. Viability of planktonic bacterial cells present in urine samples can be affected, letting the viability of biofilm-embedded bacteria unaffected, at the same time. PCR-DGGE-S's ability to detect DNA of non-living bacteria and lower culture DJCU positivity can reflect efficient antibiotic prophylaxis administration prior to intervention exclusively in patients undergoing stentation, as recommended in Guidelines (Bonkat et al., 2017; Nakada and Patel, 2017).

### Community Structure and Identified Bacterial Species in Biofilms

Focused on communities, the catheter (C) community deflection identified by culture can be explained by a different number of microbes in these communities compared to all other samples (CU, DJC, DJCU) (see Figure 1B). The community composition seemed to be influenced by the type of studied material, which corresponds to reported bacterial consortia composition differences on different materials (Paick et al., 2003; Frank et al., 2009; Holá et al., 2010; Bonkat et al., 2011, 2012b, 2013; Choe et al., 2012; Xu et al., 2012; Kliś et al., 2014). This held true much more for PCR-DGGE-S than for culture (see Figure S2), what makes us assume that PCR-DGGE-S detects consortia composition more reliably than culture. This may be caused by culture's limited capability to detect Gram-positives as mentioned above.

Regarding patients' diagnosis, we observed a certain agents significant association only with urolithiasis. Higher *Klebsiella* spp. prevalence in patients with renal calculi was not surprising, because of its urease-producing properties (Mufarrij et al., 2012; Barr-Beare et al., 2015). Interestingly, accompanying a higher *G. vaginalis* prevalence corresponded to (Schwaderer and Wolfe, 2017) finding, who demonstrated its co-detection with enterobacteria in calculi, suggesting its potential role in calculi formation to be further studied. Further, *Enterococcus* sp. was less represented in our patients with urolithiasis. The importance of non-urease-producers such as *Enterococcus* spp. remains unclear, although its decreasing effect to hyperoxaluria and calculi formation was earlier suggested (Lieske et al., 2010).

Catheter colonization is usually caused by fecal, perineal or genital microbiota (Frank et al., 2009; Holá et al., 2010; Xu et al., 2012). Representatives detected on DJCs are almost the same, just Gram-positive cocci and non-fermenting Gram-negative rods dominate over Enterobacteria (Paick et al., 2003; Bonkat et al., 2011, 2012b). Our culture results were consistent with these observations, typical known representatives were cultured. According to assumptions, higher species richness was observed in urinary catheters than DJCs.

As expected, molecular methods revealed many anaerobes, fastidious and uncommon bacteria on top of the culture with a higher Gram-positive bacteria portion. Detected uncommon bacterial species correspond with many reported in the literature (Domann et al., 2003; Azevedo et al., 2017; Shrestha et al., 2018): *Peptoniphilus, Anaerococcus, Finegoldia, Porphyromonas*, or *Veillonela* are supposed to potentially cause infections. Other condition-related pathogenic species like bifidobacteria, *Gardnerella* sp., *Varibaculum* sp. *Atopobium* sp., *Leptotrichia* sp., *Actinotignum* sp., *Propionimicrobium* sp., were detected as reported before (Domann et al., 2003; Imirzalioglu et al., 2008; Frank et al., 2009; Choe et al., 2012; Xu et al., 2012; Shrestha et al., 2018). Unexpectedly, *A. schaalii* was the second most prevalent species detected by molecular techniques. This emerging opportunistic pathogen is supposed to be the causative agent of various types of infections linked to the urinary tract (Lotte et al., 2016). *P. lymphophilum*, reported as bacteraemia co-agent together with *A. schaalii* in the catheterized patient (Ikeda et al., 2017), was another uncommon species exclusively identified by PCR-DGGE-S. Because of simultaneous significant co-detection of these species (*p* < 0.01, Fishers' test), we hypothesize their mutual relationship in the urinary tract. The role of uncommon bacteria is underestimated, in general, although they may significantly contribute to the pathophysiology and antimicrobial susceptibility pattern of CAUTI-associated biofilms as well as non-infective diagnoses (Shrestha et al., 2018). Frequent rare species exposure can lead to chronic inflammation and is hypothesized to contribute to cancer development. Specifically, *A. schaalii* has been reported as the inflammation inducing bacteria, and *P. lymphophilum* has been more commonly detected in patients with cancer biopsy (Shrestha et al., 2018).

Besides that, some other bacteria appeared significantly more frequently with each other. The presence of *G. vaginalis* in both healthy and unhealthy individuals brings ambiguousness into its virulence potential. Several studies showed the importance of *G. vaginalis* involvement with other bacteria in biofilm-based communities (Teixeira et al., 2012; Castro et al., 2019). Contrary to our results, the antagonistic relation of *G. vaginalis* and *Lactobacillus* spp. has been noted, however this relation was strain-specific, dependent on particular strain properties (Teixeira et al., 2012). In addition, we observed an inverse prevalence of *G. vaginalis* and *Enterococcus* spp., although *G. vaginalis* virulence was shown to be enhanced when present together with *E. faecalis* in dual biofilm (Castro et al., 2019). On the other hand, *E. coli* and *E. faecalis* were proved to have a synergistic effect on virulence (Lavigne et al., 2008), confirmed also in catheter-associated urinary tract infections (Tien et al., 2017), which is in concert with our findings. We observed another significant co-occurrence in the case of *F. nucleatum* and *Streptococcus* spp. Their mutually advantageous relation is suspected, because *F. nucleatum* has been reported to enhance streptococcal invasiveness by gaining entry into epithelial cells (Edwards et al., 2006) and adhering to streptococci facilitated *F. nucleatum* integration into microbial communities of the oral cavity (He et al., 2012).

Moreover, we found some bacteria (*E. coli, Klebsiella* spp., *Proteus* spp., *Enterococus* spp., coagulase-negative *Staphylococcus* spp.) more likely to be present in polymicrobial rather than monomicrobial contexts. I.e. some *Enterobacteriaceae* species are known for their non-competitive nature, thus occurring in a mixed consortium can be beneficial to them (Alteri et al., 2015; Armbruster et al., 2017).

## Conclusion

It becomes imperative that the clinicians know the biofilm composition to select the adequate therapy for effective prevention or treatment of urinary tract infections (Azevedo et al., 2017). Regardless the material, we confirmed the presence of bacteria in concentrations under the currently accepted clinical relevance threshold, but still having pathogenic potential. Bacterial colonization did not probably cause infection in our patients, nor was it considered as a reason for antibiotic treatment in them. Still device colonization may importantly influence the patients' recovery, prolong the hospitalization length, impede clinical management, and increase the risk of CAUTI (Hooton et al., 2010; Azevedo et al., 2017). This holds true particularly for immunocompromised patients. We performed the most extensive NGS analysis of catheter-related materials and at the same time the most extensive catheter-oriented non-NGS molecular study. Broad-range molecular testing of urine and sonication fluids demonstrated a good analytical performance and was shown to contribute significantly to biofilm-related bacterial consortia assessment, proposing Gram-positive's importance both in DJC and C as colonizing flora and also proving the presence of less common bacteria. Lower sensitivity but higher specificity makes PCR-DGGE-S beneficial not only for deciphering infectious etiology in cases of fastidious and difficult to culture bacteria but also as a complementary method to culture techniques for studying urinary tract associated biofilms in immunocompromised patients or other patients with a high risk of urosepsis. Its benefit was proven especially in DJCU analysis. Adapting NGS techniques for routine praxis is going to further improve diagnosis soon.

## Data Availability

All datasets generated for this study are included in the manuscript and/or the supplementary files.

## Author Contributions

IK and TF wrote the manuscript with contribution of comments from HO and BM. IK and HO provided PCR-DGGE-S analysis. IK and HO interpreted data from culture and PCR-DGGE-S. IK provided statistical analyses. BM, VH, FR, and TF designed the study. PV and BZ provided NGS analysis and bioinformatics data analysis, and contributed to PCR-DGGE-S and culture data visualization. TP, MD, VH, and FR provided culture results. PK and PT provided samples and clinical comments. All authors revised the manuscript.

### Conflict of Interest Statement

The authors declare that the research was conducted in the absence of any commercial or financial relationships that could be construed as a potential conflict of interest.

## Footnotes

^1^Available online at: http://www.sepsitest-blast.de/en/index.html

^2^Available online at: http://www.microbiology.hku.hk/16SpathDB/main.php
