# Supplementary material for: Molecular Techniques Complement Culture-Based Assessment of Bacteria Composition in Mixed Biofilms of Urinary Tract Catheter-Related Samples
Source: Front Microbiol. 2019 Mar 20;10:462. doi: 10.3389/fmicb.2019.00462 (PMC6435596; doi:10.3389/fmicb.2019.00462)
Supplement: Supplementary file 1 [file Table_1.pdf]

Supplemental material

Molecular techniques complement culture-based assessment of bacteria composition in mixed biofilms of urinary tract catheter-related samples

Table S1: Co-occurrence of bacterial species/genera

	Enterococcus spp.	*Kocuria* spp.	*E. coli*[a]	*G. vaginalis*[b]	*A. schaalii*[c]	*Campylobacter* spp.	*Streptococcus* spp.	*Lactobacillus* spp.
Corynebacterium spp.	*	**						
Enterococcus spp.			*	*				
P. lymphophilum[d]					**	**		
F. nucleatum[e]							***	
Gardnerella vaginalis								***

*** p<0.0001; ** p<0.001; * p<0.01; exclusive occurrence is marked in red; mutually higher co-occurrence is marked in green.

[a] *E. coli*: *Escherichia coli*; [b]*G. vaginalis*: *Gardnerella vaginalis*; *A. schaalii*: *Actinotignum schaalii*; [d]*P. lymphophilum*: *Propionimicrobium lymphophilum*; [e]*F. nucleatum*: *Fusobacterium nucleatum*.

Figure S1: Shannon index regarding clinical material and analytical method

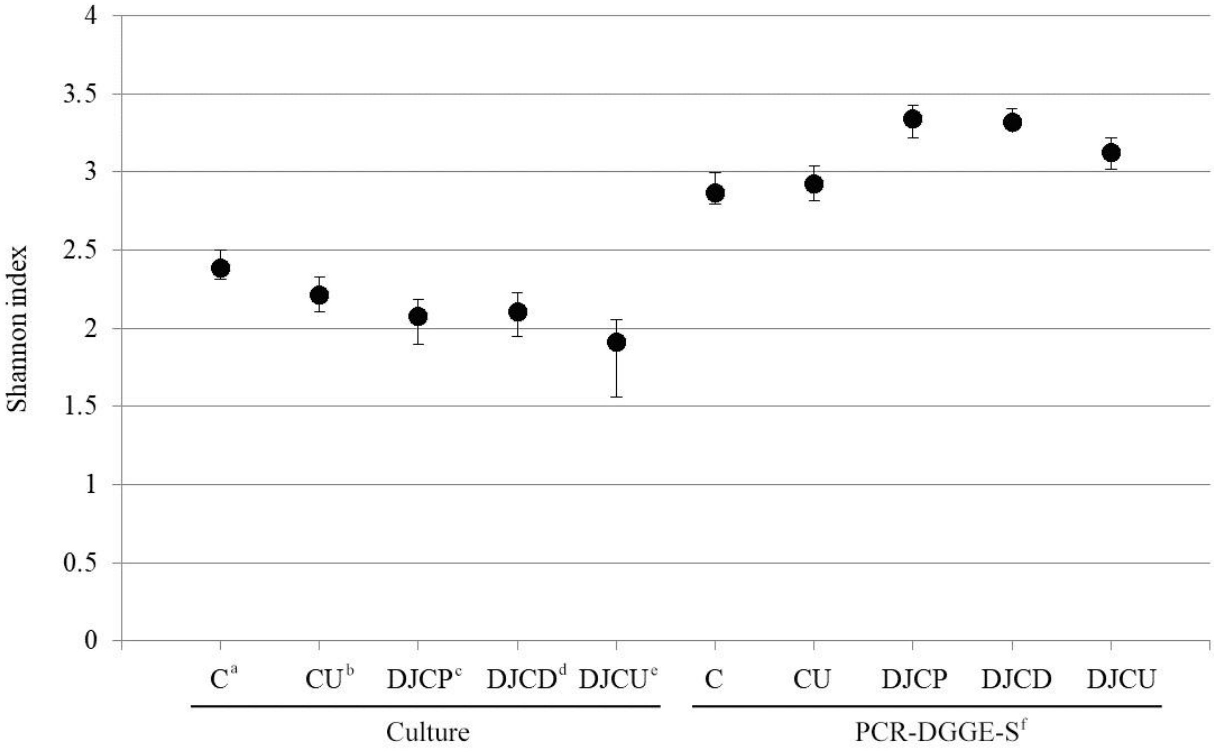

Figure shows Shannon index regardering clinical material and analytical method. Higher Shannon index is obvious in results of PCR-DGGE-S ($p<0.01$). Error bars indicate 95% confidence interval. [a]C: catheters; [b]CU: catheter urine; [c]DJCP: proximal tip of double-J catheter; [d]DJCD: distal tip of double-J catheter; [e]DJCU: double-J catheter urine; [f]PCR-DGGE-S: polymerase chain reaction, denaturing gradient gel electrophoresis, Sanger sequencing.

Figure S2: Multivariate analysis of results regarding sample material

A. PCA variance-covariance biplot for culture results

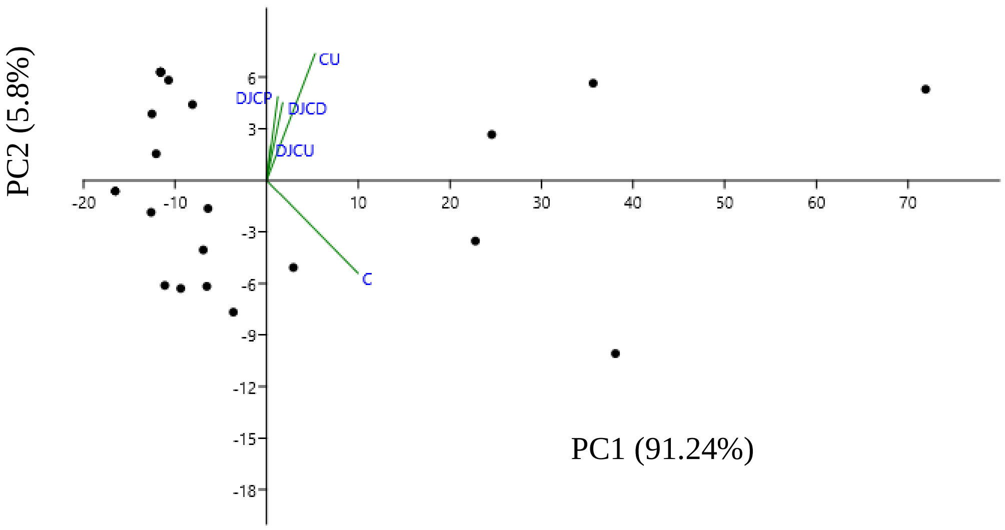

B. PCA variance-covariance biplot for PCR-DGGE-S results

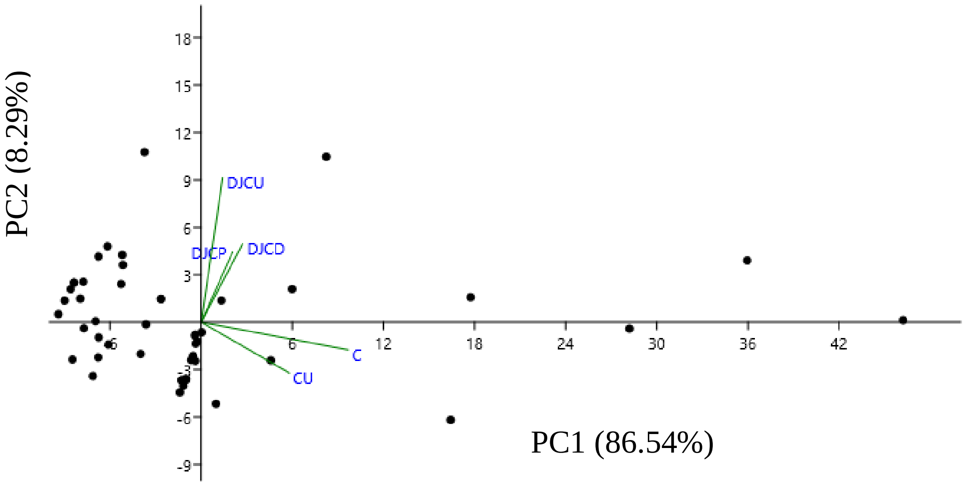

C: catheters; CU: catheter urine; DJCP: proximal tip of double-J catheter; DJCD: distal tip of double-J catheter; DJCU: double-J catheter urine.

Both biplots are results of PCA performed on the OTUs-trait (material type) matrix. Dots represent OTUs, green lines indicate the effect of the material type on the axes. Both biplots are dominated by C communities. A proximity to PC1 or PC2 is not apparent in culture results (A.), while non-dominant DJC-related materials are proximal to PC2 axis (explaining just 5.8 % of a variance). PCR-DGGE-S biplot (B.) showed C with PC1 proximity (explaining 86.54 % of a variance) and DJC related material proximity with PC2 (together explaining 94.83 % of a variance).

Table S2: Comparison of results in J=0 samples (n=10)

Representative	1	2	3	4	5	6	7	8	9	10
Actinotignum schaalii	22.5		7.6	6.7	2.7	1.7	9.4	7.1	8.6	26.9
Escherichia coli	0.1*	0.5*	0.6*	1.6*	0.2*	17.4*	3.1*	1.2*		1.9*
Enterococcus faecalis	5.9+0.4*+0.4&			4.5	0.2	13.9	17.8+1*	2.7	2.2	
P. lymphophilum[a]	0.2*			3.5*	0.6*	1.2*		2.4*		
Proteus mirabilis	1.1+0.1*		0.3*	11.2*		0.6+45.6*	19.9*	0.3		
Enterobacteriaceae		4.1	0.3	0.1	0.6	2.9		0.6		
Klebsiella pneumoniae		0.2*	5.7*							
F. nucleatum[b]			55.9*+0.1#	22.4+0.1#	68.2*		9.9*	10.4*	2.5*	
Morganella morganii				9.1*	3.3*	8.1*	17.8*	26.6*		13.4*
Fastidiosipila sanguinis	11.9*				0.6*		1.6*	1.8*		3.8
C. urealyticus[c]				2.6	2.1		3.1	5.3	0.5	
Peptoniphilus sp.				1.5	0.8		2.6	6.2	62.1	
Anaerococcus sp.					1.6		4.7	3.5	2.7	1.9
Proteus vulgaris					1.2*			22.5*		51.9*
Enterobacter sp.		86.2	0.2		0.4			0.3		
Veillonela sp.			4.4	13.0		4.6	0.5	0.9		
Parvimonas sp.			1.3	4.7	0.4		2.6	1.5		
P. somerae[d]				0.2*	7.4*		3.1*	2.1*	3.2*	
Aerococcus urinae	55.3*									
A. radicidentis[e]		8.7*								
Streptococcus anginosus			* 0.4+19.8*							
Streptococcus sp.				2.1					1.3	
Enterobacter aerogenes						0.6*				
Alcaligenes faecalis								0.6*		
Serratia marcescens										
Prevotella loeschii										
Staphylococcus aureus										
Klebsiella oxytoca										
Moraxella sp.										
Eikenella sp.					5.3		0.5	2.7		
Aerococcus sp.			1.0			2.9				
Corynebacterium sp.			0.2				2.1			

Neisseria sp.					4.3			1.8		
Facklamia sp.	2									
Pseudomonas sp.		0.2								
Prevotella bivia			0.1							
Bergeyella sp.			1							
Howardella sp.			1							
Bifidobacterium breve				1.2						
Bacteroides sp.				9.9						
Peptostreptococcus sp.				0.3						
Providencia sp.				5						
Planomicrobium sp.						0.6				
Ezakiella sp.									14	
Peptococcus sp.									1.3	
Snaethia sp.									1.6	
Other not determined				0.1						
**In total**										
Culture	3	1	0	5	5	4	0	6	4	4
PCR-DGGE-S[f]	3	3	4	2	3	3	6	3	3	2
NGS[g]	8	6	15	18	17	11	15	20	11	6

[a]*P. lymphophilum*: *Propionimicrobium lymphophilum*; [b]*F. nucleatum*: *Fusobacterium nucleatum*; [c]*C. urealyticus*: *Campylobacter urealyticus*; [d]*P. somerae*: *Porphyromonas somerae*; [e]*A. radicidentis*: *Actinomyces radicidentis*; [f]PCR-DGGE-S: polymerase chain reaction, denaturing gradient gel electrophoresis, Sanger sequencing; [g]NGS: next-generation sequencing (*16S rRNA* amplicon Illumina sequencing).

Table shows comparison of results in samples with completely discrepant results between culture and PCR-DGGE-S (J = 0, n = 10). Results of culture are in blue; result of PCR-DGGE-S in red, and result of NGS in green. *indicates genus determination; & indicates family determination; # indicates order determination. More numbers in tile indicates identification to different taxonomic levels, according to symbols. If no symbol is applied, identification corresponds to the taxonomic level of the first column. Relative abundances (%) of NGS reads are shown in NGS dedicated

columns. Fourteen framed representatives were not confirmed by NGS (detected solely by culture or PCR-DGGE-S). Not determined representatives were not included in total counts.

Table S3: Comparison of results in 0.25≤J≤0.5 (n=9) samples

	J = 0.25 (n = 5)					J = 0.5 (n = 4)			
Representative	1	2	3	4	5	6	7	8	9
Escherichia coli	27.2*	0.1*	7.5*	72.9*	0.8*	21.2*	0.4*	3.1*	32.1*
Enterococcus faecalis	0.4*		10+1.1*	0.9	3.7+0.2*	31.9+0.5*	18.1+0.2*	14.2+0.3*	0.6
Proteus mirabilis		76.8	0.1+3.0*		3.8*	0.6+9.9*		0.3+5.7*	0.6+13.5*
Actinotignum schaalii	5.0			* 9.3	1.8+1*	2.6*	4.2*		* 0.6*
Aerococcus urinae	45.4*			7.5*	34.5*				
Campylobacter urealyticus			27.4			17			19.9
Peptoniphilus sp.			4.9			3			0.6
Fastidiosipila sanguinis					49.7*	1*		4.6*	
P. lymphophilum[a]			18*						
Pseudomonas aeruginosa								1.4+2.6*	
Proteus vulgaris		3.7*							
Staphylococcus haemolyticus			*[b] 0.1*[b]						
Staphylococcus chromogenes			*[b] 0.1*[b]						
Providencia rettgeri								* 7.7*	
Staphylococcus aureus								29.9*	
Citrobacter freundii							*		
Streptococcus agalactiae									
Staphylococcus epidermidis									
Klebsiella oxytoca									
Klebsiella pneumoniae									
Serratia marcescens									
Kingella kingae									
Staphylococcus capitis									
Raoultella sp./Citrobacter sp.									
Enterobacter sp.	0.8		1.2	2.8		0.2	5.7	1.7	1.2
Enterobacteriaceae	0.4	0.1	0.1		0.1	0.1	6.5	0.9	0.6

Anaerococcus sp.		0.1			0.3	5.8		9.1	1.2
Planomicrobium sp.						0.7	1	0.3	
Providencia sp.			20			5.4			22.9
Veillonela sp.	20.5			6.5					
Alcaligenes sp.		5.1						0.3	
Streptococcus sp.			6.2					0.3+12.8*	
Bifidobacterium sp.	0.4								
Atopostipes sp.		12							
Oligella sp.		0.1							
Paenalcaligenes sp.		0.8							
*Corynebacterium*sp.		1.2							
Morganella sp.					3.8				
Parvimonas sp.						0.4			
Kluyvera sp.							63.5		
Staphylococcus sp.							0.2		
Actinomyces sp.								4.8	
Other not determined									
**In total**									
Culture	6	4	6	2	5	3	2	6	3
PCR-DGGE-S[c]	4	1	4[d]	3	5	3	4	3	3
NGS[d]	8	10	11[d]	6	9	13	8	14	10

[a]*P. lymphophilum*: *Propionimicrobium lymphophilum*; [b]PCR-DGGE-S and NGS detected coagulase-negative staphylococcus and *Staphylococcus* sp., respectively. Strain determination was not possible. We match both coagulase-negative staphylococci detected by culture, but in the sum just as one representative is counted. [c]PCR-DGGE-S: polymerase chain reaction, denaturing gradient gel electrophoresis, Sanger sequencing; [d]NGS: next-generation sequencing (*16S rRNA* amplicon Illumina sequencing).

Comparison of results among culture, PCR-DGGE-S, and NGS in samples with 0.25≤J≤0.5 (n=9). Results of culture are in blue; result of PCR-DGGE-S in red, and result of NGS in green; *indicates genus determination. Relative abundance (%) of sequenced reads is showed in NGS dedicated columns. More numbers in tile indicates identification to different taxonomic levels, according to symbols. If no symbol is applied, identification corresponds to the taxonomic level of the first column. Not determined representatives were not included in total counts. Fifteen framed representatives not confirmed by NGS (detected solely by culture or PCR-DGGE-S) are framed.

Table S4: Comparison of results in J=1 (n=10) samples

Representative	1	2	3	4	5	6	7	8	9	10
Enterococcus faecalis	12.5+0.5*	9.0+0.6*	10.2+0.5*	27.7+1.3*	92.4+4.3*	0.7	2	69.1+7.2*	0.9	8.1+0.3*
Escherichia coli		90.1*				98.9*	97.1*		97.4*	2*
Proteus mirabilis			86.2+0.4*							0.8+3.4*
Pseudomonas aeruginosa								11,8+1.4*		10.1+20.9*
Staphylococcus aureus				0.3+70.4*						11.2*
Klebsiella pneumoniae	82.6*									
Staphylococcus epidermidis					0.1+0,5#					
Providencia rettgeri										29.6*
Proteus vulgaris										
Klebsiella oxytoca										
Enterobacter sp.	2.3	0.2			0.1	0.2	0.6		1.7	1.7
Enterobacteriaceae	2.1		1.3				0.4			0.6
Anaerococcus sp.			1.3		0.5			4.4		1.7
Peptoniphilus sp.					0.3			0.1		
Fastidiosipila sp.								0.6		1.1
Campylobacter sp.					0.1					
Planomicrobium sp.					0.1					
Clostridiales					0.1					
Porphyromonas sp.					0.2					
Prevotella sp.					0.5					
Ezakiella sp.					0.7					
Aerococcus sp.								0.8		
Actinotignum schaalii								1.9		
Staphylococcus sp.								2.1+0.1#		
Finegoldia sp.								0.1		
Streptococcus sp.										2.5
Alcaligenes sp.										5.3

Actinomyces sp.										0.6
Kluyvera sp.										0.3
**In total**										
Culture	2	2	2	2	2	2	2	1	2	6
PCR-DGGE-S[a]	2	2	2	2	2	2	2	1	2	6
NGS[b]	4	3	4	2	11	3	4	9	3	14

[a]PCR-DGGE-S: polymerase chain reaction, denaturing gradient gel electrophoresis, Sanger sequencing; [b]NGS: next-generation sequencing (*16S rRNA* amplicon Illumina sequencing).

Table shows results in samples with completely concordant results of culture and PCR-DGGE-S (J=1, n=10). Results of culture are in blue; result of PCR-DGGE-S in red, and result of NGS in green; *indicates genus determination; & indicates family determination; # indicates order determination. Relative abundance (%) of sequenced reads is showed in NGS dedicated columns. More numbers in tile indicates identification to different taxonomic levels, according to symbols. If no symbol is applied, identification corresponds to the taxonomic level of the first column. Two representatives not confirmed by NGS (detected by culture and PCR-DGGE-S) are framed.
